# Motivational Factors and Barriers Towards Initiating and Maintaining Strength Training in Women: a Systematic Review and Meta-synthesis

**DOI:** 10.1007/s11121-021-01328-2

**Published:** 2021-11-20

**Authors:** Aishwarya Vasudevan, Elizabeth Ford

**Affiliations:** Brighton and Sussex Medical School, Brighton, BN1 9PH UK

**Keywords:** Strength training, Women, Motivators, Barriers, Stigma

## Abstract

Strength training (ST) or resistance training is important in the development and maintenance of musculoskeletal and cardiovascular health in women of all ages; however, uptake of ST amongst women is low. To improve female musculoskeletal health, it is vital that more women are encouraged to participate in ST to maintain musculoskeletal integrity. This systematic review aimed to identify motivators and barriers to women initiating and maintaining ST. Following protocol registration and systematic search, studies were included if they were primary qualitative or mixed-method studies reporting participant verbatim quotes, included adult women, and focused on motivators and barriers for ST. Searches generated 2534 articles from 3 databases, with 20 studies (*N* = 402 participants) meeting eligibility criteria. Participant quotes and authors’ interpretations were analysed using thematic synthesis. The most frequently observed barriers were gender-based stigmas, discouragement, and negative comments, particularly in women currently engaging in ST. Other factors associated with poor adherence included boredom, poor knowledge of ST, poor gym accessibility, lack of supervision or routine, and difficulty in balancing work and family life. Social support from friends and family, words of affirmation, and accompaniment facilitated ST, particularly in older women. Women who saw expected results such as weight loss were motivated to continue ST. Interventions aimed at increasing participation in ST amongst women should focus on the specific benefits valued by women and the dissemination of accurate information to counter misconceptions and increase knowledge. The adaptation of gym environments to make them more welcoming to women, and reduce gender-focused criticism, is especially important.

## Introduction

Strength training (ST) (or resistance/weight training) is a form of physical activity that improves muscular strength by training a muscle (group) against external resistance. This may involve using free weights, resistance machines or bands, bodyweight exercises, or daily activities such as lifting, carrying, using stairs, wheelchairs, and gardening (Gibson-Moore, 2019).

Lean muscle mass has been shown to decrease every decade between 3 and 8% after 30 years and 5–10% after 50 years in both men and women (Volpi et al, 2004). Maintaining muscle mass is essential in preventing deterioration of musculoskeletal health and development of metabolic syndrome (a combination of high blood glucose, cholesterol, lipids, blood pressure, and central obesity) (Broeder et al., 1992). ST not only increases muscular strength, endurance (Westcott et al., 2009), and helps prevent metabolic syndrome (Broeder et al., 1992) but also reduces body fat (Strasser & Schobersberger, 2011), improves resting metabolic rate (Broeder et al., 1992), symptoms of arthritis, physical function (Mayer et al., 2011), and mental health (Nikfarjam et al., 2015). To improve and maintain musculoskeletal health, the Department of Health and Social Care in the United Kingdom (UK) recommends that all adults engage in ST involving major muscle groups in the upper and lower body at least twice a week (Gibson-Moore, 2019).

ST is particularly beneficial for women. Post-menopausal hormonal changes put women at a greater risk of younger onset of bone loss and developing osteopenia, osteoporosis, osteoarthritis, more severe osteoarthritis, and other musculoskeletal conditions compared to men (Ji & Yu, 2015; Alswat, 2017; Srikanth et al., 2005). These conditions place a heavy burden on the UK’s National Health Service (NHS). In 2010, £5.5 billion was spent just on community, social services, and hip and knee replacements for osteoarthritis and fragility fractures due to osteoporosis (National Institute for Health and Care Excellence (NICE), 2020; NHS RightCare, 2017). ST increases bone mineral density and reduces risk of osteoarthritis, osteoporosis, and osteopenia (Going & Laudermilk, 2009). Thus, there is a pressing need for health promotion strategies encouraging women to engage in ST to delay the onset of such conditions.

In the UK, in 2016, only 23% of women over 16 years met national ST or aerobic exercise guidelines (NHS Digital, 2017); however, it should be noted that this is self-reported data, and therefore, the percentage meeting physical activity guidelines are likely much lower. There is also a lack of separate data on the percentage of women meeting ST guidelines, which are less often met than aerobic exercise, so the true percentage of women engaging in ST is likely even lower. Haines et al. (2008) identified that the ratio of men to women using free weights in gyms was 27:1 and Dworkin (2003) also recognised that women predominantly use cardiovascular machines over ST equipment as a result of strong gender-based stigmas. Training habit and confidence with weights or machines are built over time and early intervention, and commencement of strength training is beneficial for developing and maintaining muscle mass, strength, and bone density in adult women throughout life (Gardner et al., 2012). ST may have specific motivators and barriers in women that differ from other forms of physical activity (Burton et al., 2017). These factors may also vary considerably between age groups, socio-cultural backgrounds, and individuals with disabilities or disease.

Many studies have previously identified correlates, barriers, and motivators of ST in men and women. Demographic determinants associated with ST participation identified by previous research include being male, increasing education, younger age, and Caucasian background, with the strongest predictors of participation being involvement in other physical activity and perceived good health (Chevan, 2008; Loustalot et al., 2013; Galuska et al., 2002). Barriers to ST in women reported by quantitative studies include time consumption, tiredness, appearing weak, silly or uncoordinated, fear of looking ‘big and bulky’, muscle soreness, judgement by friends, discomfort doing ST alone, around men or in crowded gyms, poor previous experience or failure, lack of knowledge of ST, level of discipline required, and desire (Peters et al., 2019; Hurley et al., 2018; Salvatore & Marecek, 2010). Motivators identified by previous research include making ST part of a routine, gaining supervision, enjoying ST, and finding ST helps with weight management and physical and mental well-being (Viljoen & Christie, 2015; Harne & Bixby, 2005). These were all single quantitative studies where participants endorsed researcher-proposed barriers and motivators.

Previous reviews have attempted to synthesise results of several studies. Key systematic reviews have identified motivators and barriers of ST specifically in older adults (Burton et al., 2017; Cavill & Foster, 2018; Taunton et al., 1997), finding that not having time, a perceived risk of inducing a heart attack or stroke, and worries about getting too muscular were particular barriers in the older age group. A review looking at all adults (Rhodes et al., 2017) found that low education levels and perceived health status were consistent barriers to ST. Reviews which have studied factors of engagement in any physical activity in women (rather than ST alone) (Babakus & Thompson, 2012; Resurrección et al., 2017; Harrison et al., 2018) have found that for women, taking time out of domestic duties can be seen as selfish. Some women feel uncomfortable in mixed-sex facilities and cite religious reasons or a lack of family or community support as barriers.

While these barriers may be relevant for any physical activity, there remains a gap for the synthesis of qualitative literature elucidating specific barriers and motivators for strength training in adult women. A meta-synthesis bringing together qualitative studies in a structured manner will highlight key themes and factors associated with ST participation and has the potential to capture themes missed by quantitative studies (Snilstveit et al., 2012), as qualitative studies allow the chance to hear women’s voices about this topic. A comprehensive understanding of ST barriers and motivators from women’s perspectives will enable delivery of effective interventions help to improve ST environments and programmes to make them more appealing to women and thus improve women’s uptake of this important physical activity.

## Methods

The reporting of this review conforms to the Preferred Reporting Items for Systematic Reviews and Meta-Analyses (PRISMA) guidelines (Liberati et al., 2009) and Enhancing Transparency in Reporting the Synthesis of Qualitative Research (ENTREQ) guidelines (Tong et al., 2012). The study protocol was also registered in advance on PROSPERO (CRD42020157351).

### Information Sources and Search Strategy

**Fig. 1:**
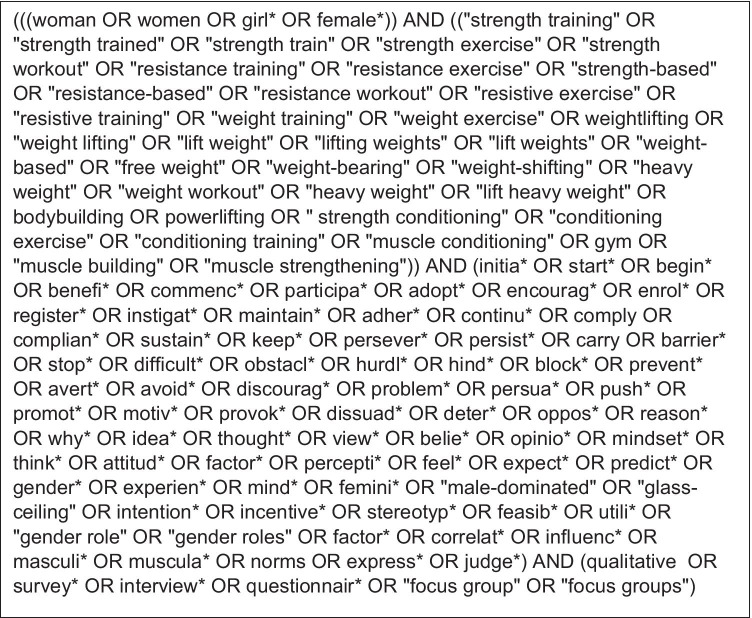
Example of one full search strategy

Three databases (PsychINFO, PubMed, and ASSIA) were searched for articles between 7th November 2019 and 4th February 2020, and again on 9th September 2021 using a pre-planned search. Only peer-reviewed published articles were searched. Other search strategies involved tracking citations of eligible studies (forward-searching) and examining reference lists of eligible studies (reverse searching). Subject headings and truncated keywords used to search titles and abstracts of papers were in relation to four concepts: women, ST, motivators and barriers, and qualitative research. When conducting the searches, search terms were combined using Boolean terms ‘OR’ and ‘AND’. The full search strategy is depicted in Fig. 1.

The limits used and search dates for the first systematic searches were (limits were replicated for the second search):

1. PsycINFO (7/11/2019) (limits: females; peer-reviewed; English language)
2. PubMed (01/12/2019) (limits: females; English Language)
3. ASSIA (08/12/2019) (limits: peer-reviewed; adults (> 18 years old); English language; document type: article, evidence-based healthcare, interview, literature review, report, review, transcript; source type: reports, scholarly journals)

## Study Selection

All search results were downloaded into Mendeley. Studies were selected in 3 stages. In stage 1, one author (AV) scanned all titles and excluded any studies that did not meet the inclusion criteria which identified 2176 papers as not relevant. Stage 2 involved AV screening 358 abstracts and excluding any studies not meeting the inclusion criteria. After removal of duplicates and inspecting abstracts, stage 3 involved scrutinising 64 full-text articles (21 articles from forward and reverse searches) by two authors (AV and EF) to confirm that all papers met all eligibility criteria. Where there was disagreement, AV and EF discussed whether the article met all inclusion criteria until there was agreement.

### Eligibility Criteria

The review was limited to studies meeting the following eligibility criteria using the SPIDER parameters from Cooke et al. (2012).

1. Sample: adult women > 18 years, women’s opinions should be clearly separate from male opinion.
2. Phenomenon of interest: studies explored participation in/continuing ST training and if ST was clearly separate from other forms of physical activity. ST refers to any exercise intervention involving external resistance with either the aim of building muscle mass or improving muscle strength. Women may have current or previous ST experience and have any health condition.
3. Design: interviews, focus groups, mixed-methods studies, and surveys with open text responses, with quotes reported verbatim.
4. Evaluation: motivational factors and barriers to starting or maintaining ST.
5. Research type: primary qualitative research in English language only. ‘Qualitative’ was broadly defined as any results reporting as text rather than numbers. Mixed-method studies were included if they reported results analysed qualitatively.

### Risk of Bias Assessment

Since there is currently no widely agreed on quality reporting criteria for qualitative research (Atkins et al., 2008) or meta-syntheses (Dheensa et al., 2013), the Joanna Briggs Institute Checklist for Qualitative Research (Joanna Briggs Institute, 2017) was used to examine methodological quality or risk of bias in qualitative and mixed-method studies prior to data collection. One author (AV) conducted the quality assessments and reliability of these ratings for all eligible papers. Of 10 possible points, all studies scored at least 7. No studies were excluded on quality grounds.

### Data Extraction of Study Parameters

Data on author names, year of publication, country of study, number of participants, age range, average age, participant demographics, study type and methodology, objective of the study, and quality score were extracted from eligible studies into an Excel file.

### Strategy for Data Synthesis

Data synthesis was conducted in two stages using the thematic synthesis method in accordance to Thomas and Harden (2008). Stage one involved coding of text ‘line by line’ of findings from eligible studies using NVivo Software by one author (AV). Quotes verbatim were identified from findings and transferred onto the database verbatim. Verbatim text obtained from the primary studies was converted to ‘codes’ that capture the meaning and content of each sentence. If new studies had similar meaning or content, they were added to previously created codes. If participant quotes or author interpretation did not match previously created codes, new codes were created. Quotes matching more than one code were categorised using multiple codes.

Stage two involved using ‘free codes’ from stage one and organising them into ‘descriptive themes’ inductively by one author (AV). This involved identifying similarities and differences between codes and organising them into a hierarchical tree structure. If codes did not fit into a ‘descriptive theme’, these remained as free codes.

After stages 1 and 2 of data extraction, stage 3 involved the generation of ‘analytical themes’. Barriers and facilitators from the women’s views obtained from stages 1 and 2 of data extraction were inferred and the implications of their views were considered for intervention development. This was done individually by one author (AV) first and the analytical themes and any discrepancies that arose were discussed with the second author (EF). This process was repeated until new themes were sufficiently able to describe and/or explain all initial descriptive themes, inferred barriers, facilitators, and implications for intervention development. Due to lack of resources, only a single author conducted the coding; however, codes and themes were checked by the second author (EF) at each stage.

## Results

### Studies

**Fig. 2:**
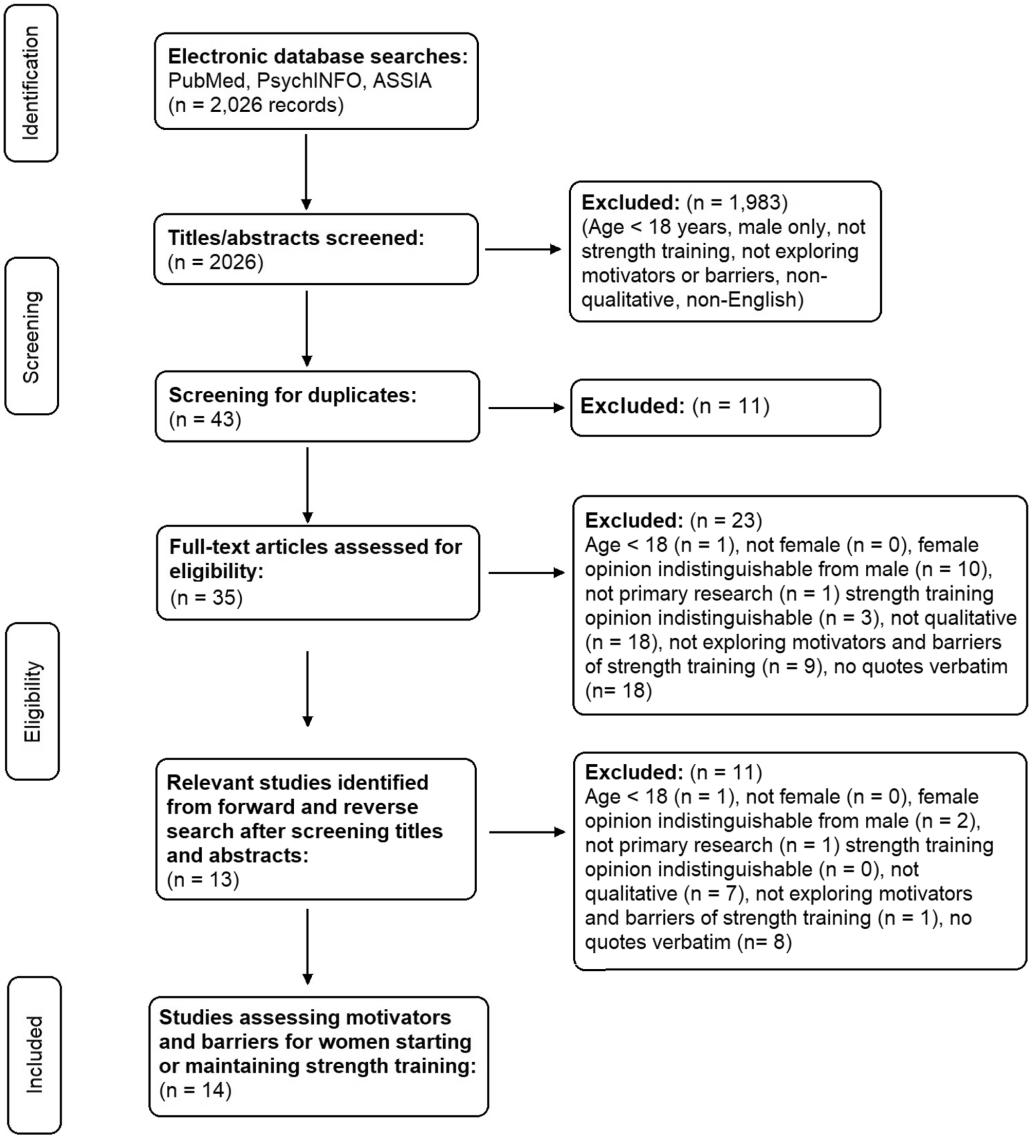
PRISMA flow chart

The systematic search returned 2534 papers. In total, 20 papers were found that met all eligibility criteria and reported on motivators and barriers for women initiating or maintaining ST (Fig. 2). Study characteristics are shown in Table 1. All studies selected originated in either the USA, UK, Australia, or Canada. Nine studies explored facilitators and barriers in women currently meeting ST recommendations. Six studies investigated motivators and barriers in specific patient groups, i.e. multiple sclerosis (MS), Parkinson’s disease, type 2 diabetes, raised body mass index (BMI), disabled individuals, and those from rural areas. Nine studies recruited participants to undertake a ST programme for various lengths of time and interviewed participants or organised focus groups about their views on the programme. Quotes from the interviews and focus groups are included.

**Table 1:** Study characteristics

**Authors (year of publication)**	**Country**	**Number of female participants**	**Age range (average age) in years**	**Participant demographics**	**Study type and methodology**	**Objectives of study**	**Quality score**
Bopp et al. (2004)	USA (South Carolina)	39	(67.5)	59% Caucasian, 41% African American 35.9% Married, 30.8% widowed, 33.3% divorced/separated 43.6% Retired, 17.9% employed, 38.5% homemaker	Qualitative Questionnaire, semi-structured interviews	To better understand factors related to ST in older African American and Caucasian women in rural areas by assessing demographic, psychological, and social influences	7/10
Brace-Govan (2004)	Australia (Melbourne)	16	15–32 (22.87)	Elite athletes 50% weightlifters (powerlifters *n* = 6, Olympic weightlifters *n* = 2) 50% weight-training athletes. All Caucasian, in professional employment, semi-skilled, students, or unemployed	Extended, in-depth interviews	To explore women’s experiences of weightlifting, self-perception, everyday interactions, and other’s perceptions of these women.	7/10
Coen et al. (2018)	Canada (district/city unknown)	34	25–64 (40)	Regular gym user holding a co-ed gym membership, engage in ST and/or cardiovascular training Mostly white Canadian, 8 ethnic-minority group/mixed-heritage Mostly heterosexual, 5 homo-sexual Socio-economically diverse (13% professional/skilled occupations, 33% clerical/technical, 20% students, 3% receiving social support/unemployment insurance) Education (35% graduate degree, 33% undergraduate degree, 23% college diploma, 10% high school degree)	Qualitative Semi-structure interviews	To explore gender disparities and differences in exercise participation in gym environments with a particular focus on how gender influences exercise practices in men and women	8/10
Dionigi (2007)	Australia (New South Wales)	6	65–72	Healthy individuals (no major illnesses, injuries, and moderately active) who were all white non-smokers, considered themselves ‘healthy’. Majority of participants had no ST experience but were involved in other recreational and sporting activities	Mixed-methods 12-week exercise program Semi-structure interviews	To determine the perceived psychological benefits of ST and understand the connection between metal well-being and exercise in older adults.	7/10
Dodd et al. (2006)	Australia (Victoria)	7	27–61 (46.8)	MS (1–9 years post-diagnosis), between normal and moderate disability, low to moderate psychological and physical impact of disease	Mixed-methods Exercise intervention, semi-structured interviews	To explore outcomes of a ST intervention programme for adults with MS and identify motivators and barriers to completion of this ST programme in MS patients.	8/10
Doherty et al. (2018)	UK (Northern Ireland)	35 (16 pre-menopausal, 8 peri-menopausal, 11 post-menopausal)	Pre-menopausal (32) Peri-menopausal (49) Post-menopausal (59)	74–78% university degree 77% post-menopausal women married 70% of women not taking part in ST 20% undertaking some ST but not reaching recommended levels of activity 10% currently undertaking recommended levels of ST	Mixed-methods Questionnaires, semi-structured interviews and focused groups	To identify factors influencing pre-, peri- and post-menopausal women’s participation in ST using the theory of planned behaviour	8/10
Fleig et al. (2016)	Canada (Vancouver)	10	(66.23)	85% with at least post-secondary education 70% retired	Mixed-methods 4-months feasibility intervention, semi-structured interview	To test the viability of a theory-based behaviour change intervention that encouraged older women to incorporate strength and balance exercises into activities of daily living.	7/10
Foyster et al. (2020)	Australia (Victoria)	13	> 50 years	Female, and currently participating in powerlifting training.	In-depth, semi-structured interviews	To explore the experiences of older women who engage in powerlifting training to identify uptake and adherence factors specific to this group.	8/10
Gilson et al. (2008)	USA (Michigan)	43	18–24 (20.26)	University collegiate athletes, all subjects engaged in structured ST 1–5 times a week	Mixed-methods Questionnaires, semi-structured interviews	To identify motivational strategies used by collegiate athletes engaging in ST to continue ST and explore the theory that 5 achievement goal orientations are consistent amongst athletes engaging in ST with the same orientation.	7/10
Gluchowski et al. (2018)	New Zealand (Auckland)	Unknown	67 ± 4.5	All participants were independent, community-dwelling (Auckland, New Zealand) adults over the age of 60. All our participants were also healthy, high functioning, physically active, and resistance-trained.	8-week training intervention Focus group discussions	Explore the thoughts and perspectives of older adults at the completion of a highly structured, very heavy-load, resistance training intervention.	7/10
Guess (2012)	UK (London)	11	(40.2)	18–30 years (*n* = 4 including 1 South Asian) > 60 years (*n* = 4) BMI > 40 kg/m^2^ (*n* = 5 including 1 South Asian) South Asian females (*n* = 6) Mean BMI 33.8 kg/m^2^ Multi-faith, multi-ethnic, multi-religious	Focus groups Semi-structured interviews	To understand reasons why individuals who were overweight or obese choose to perform aerobic or resistance exercise.	8/10
McGlashan et al. (2020)	Australia (Victoria)	6	69–78 (73.2)	All retired, previously professionals, married or widowed with sarcopenia, joint replacements, arthritis or stenosis, previously active, involved in general sports, rehabilitation, resistance training, and daily activity, currently engaged in powerlifting	Semi-structured interviews about participants’ experiences as well as researcher observations.	To conduct an evaluation of the ‘Never Too Late Programme’ for powerlifting and its impact on associated biopsychosocial health outcomes.	8/10
Nazaruk et al. (2016)	USA (Georgia)	15	20–44 (30.2)	White (10), African American (4), Hispanic (1) Married (8), single (7), has children (6) University degree (10), high school degree (5) Full-time employment (11), part-time (1), unemployed (3)	Qualitative Semi-structured interviews	To identify perceptions, knowledge, motivation, and skills regarding ST in rural women aged 20–44 and identify patterns in ST in this cohort.	8/10
O'Brien et al. (2008)	Australia (Victoria)	3	50–68 (61.3)	Parkinson’s disease (1–6 years post-diagnosis), mild symptoms	Mixed-methods Exercise intervention, semi-structured interviews	To explore beliefs of Parkinson’s disease (PD) adults on an ST intervention and identify motivators and barriers to continuing the programme.	8/10
O'Dougherty et al. (2008)	USA (Minnesota)	49	25–44	25 ethnic-minorities, 24 Caucasian BMI 25–35 kg/m^2^, pre-menopausal, sedentary or modestly physically active, non-smoking	Mixed-methods, Randomised controlled intervention trial, Focus groups	To explore factors associated with adherence to an ST intervention in women who were overweight to mildly obese, aged 25-44 years.	7/10
Richardson et al. (2017)	UK (Birmingham)	8	26–60 (43)	Mostly white British, 1 Trinidadian-British Individuals with disabilities (spinal cord injury, fibromyalgia, brittle bone syndrome)	Qualitative Semi-structured interview	To understand how having a disability affects experiences at the gym and identify motivators and barrier to exercising in gyms.	8/10
Rosenthal et al. (2021)	USA (Indiana)	23	18–22 (19.95 ± 0.97)	All current students at university majoring in either ballet or contemporary dance training competitively, studio or pre-professional with most students incorporating some form of strength training	Qualitative Semi-structured interview	To qualitatively explore perceptions and utilization of STC in collegiate contemporary and ballet dancers.	8/10
Shilling and Bunsell (2009)	UK (Canterbury)	26	23 - 48	Female bodybuilders, 17 previously competed, aiming to improve muscle size and definition attending bodybuilding gyms or public gyms regularly Occupation—middle-classes ranging from fitness instructor to office worker to university lecturer). 50% had degrees, 5 had children, and all but 2 white British (the others were black British/Afro-Caribbean).	Qualitative Semi-structured interviews	To explore the motivations and experiences of female bodybuilders themselves in explaining why they remain engaged in a male-dominated activity.	7/10
Tulloch et al. (2013)	Canada (Ottawa)	10	39–70	Previously inactive, type 2 diabetes	Qualitative Semi-structured interview	To explore motivators and barriers of aerobic, ST, or combined exercises in patients with type 2 diabetes.	8/10
Worthen and Baker (2016)	USA (Oklahoma)	29	20–57 (36)	11 Previously trained for a bodybuilding competition within the last year 86% White, 7% Black or African American, 3.5% Hispanic/Latino 31% Married, 17% divorced/separated, 38% single 54% University degree holder, 21% incomplete degree, 10% associate degree, 3.5% graduate equivalency diploma, 3.5% vocational school 93% Heterosexual, 7% bisexual	Qualitative Questionnaire, semi-structured interviews	To examine women’s bodybuilding in the context of Lyng’s (1990, 2005) edgework model (conceptualising risk-taking in order to understand what motivates people to voluntarily engage in high-risk behaviours)	7/10

### Thematic Synthesis Findings

Six different themes emerged from the 20 eligible papers. These themes were split between barriers and motivators with 4 themes fitting into both barriers and motivators. Twelve sub-themes in barriers and eight sub-themes in motivators were identified (Table 2).

**Table 2:** Themes and sub-themes identified

**Barriers**	**Motivators**
•Social: -Gender-based and social stigma -Lack of support •Psychological: -Lack of progression or unexpected results -Interest, personality, and preference •Knowledge: -Inadequate knowledge and understanding of ST -Misconceptions and gender-based stigmas •Physical: -Injury, pain, and lack of fitness •Gym infrastructure and financial barriers: -Facilities and accessibility -Financial barriers -Lack of supervision or instruction •Time-effort: -Availability of time and work-life balance -Family commitments and other obligations	•Social: -Camaraderie in training -Support from friends, family and significant others •Psychological: -Body image and progression -Obligatory •Knowledge: -Improvement in general health and musculoskeletal health -Increased knowledge and Understanding of ST •Gym infrastructure and financial barriers: -Financial incentives -Supervision and instruction

### Barriers

The papers contributing to each barrier theme are shown in Table 3.

**Table 3:** How each study contributed to each theme—barriers

	**Social**	**Psychological**	**Gym infrastructure and financial barriers**	**Physical**	**Knowledge**	**Time-effort**	**Previous ST**
Bopp et al. (2004)	Yes		Yes	Yes	Yes		None
Brace-Govan (2004)	Yes		Yes		Yes		Elite athletes
Coen et al. (2018)	Yes	Yes					Regular ST
Dionigi (2007)		Yes		Yes	Yes		No ST (otherwise active)
Dodd et al. (2006)			Yes	Yes		Yes	Unknown
Doherty et al. (2018)	Yes	Yes	Yes	Yes	Yes	Yes	70% no ST 20% some ST 10% regular ST
Fleig et al. (2016)		Yes			Yes	Yes	Unknown
Foyster et al. (2020)	Yes		Yes		Yes		Regular ST
Gilson et al. (2008)							Structured ST up to 5× per week
Gluchowski et al. (2018)						Yes	ST intervention
Guess (2012)	Yes	Yes	Yes		Yes		Unknown
McGlashan et al. (2020)				Yes	Yes	Yes	Regular ST
Nazaruk et al. (2016)	Yes		Yes	Yes	Yes	Yes	Unknown
O'Brien et al. (2008)							Unknown
O'Dougherty et al. (2008)	Yes	Yes	Yes			Yes	Sedentary–modestly active
Richardson et al. (2017)	Yes		Yes				Unknown
Rosenthal et al. (2021)	Yes	Yes			Yes		Regular ST
Shilling and Bunsell (2009)	Yes						Regular ST
Tulloch et al. (2013)				Yes		Yes	No ST (sedentary)
Worthen and Baker (2016)	Yes						Regular ST

### Social Barriers

#### Gender-based and Social Stigma

The main social barriers that arose across studies were gender-based and social stigmas. Many women, who were not currently engaged in ST, expressed barriers associated with appearance, particularly, not wanting to ‘look like a man’(Bopp et al., 2004). Dancers emphasised the highly aesthetic nature of their sport not wanting to ‘build bulky muscles’, being discouraged by their instructors (Bopp et al., 2004).

ST women also identified multiple deterrents within gyms that discouraged them from ST, particularly the comments received from others.

‘I’ve had strangers come right up to me in the gym and just say, “You’re a woman, women shouldn’t be muscular. Female bodybuilders look disgusting”, “She looks like a man”, and “If you carry on training like that you’ll look like a man”.’ (Bopp et al., 2004)

ST women also identified the segregation of cardiovascular and ST equipment at the gym prevented women from crossing ‘the line’. Women-only gyms had a significant lack of ST equipment which was perceived as ‘women don’t need those weights’ (Coen et al., 2018).

‘A separation where women are supposed to be and where men are supposed to be’. (Coen et al., 2018)

If women did ST, they felt perceived as a ‘princess’ or as ‘cardio bunnies’ (Coen et al., 2018) if they used cardiovascular machines. Some women even ‘hate’ using cardiovascular equipment as a result.

Women also perceived that men were disproportionately ‘taking up space’, tended to ‘hog a piece of equipment’, and were ‘grunting and groaning’ in ST sections of the gym which left women feeling crowded out or not ‘want to stick around as long’ (all quotes from Coen et al., 2018). This was also highlighted by Bopp et al. (2004).

‘…I’ve tried lifting in other gyms before, over the years-I always found they’re full of these gross men grunting and carrying on…Um, well, uh, he [coach] reassured me about the atmosphere in the gym, that it wasn’t that grunting groaning sort of making, basically making women feel unwanted in the gym.’ (Bopp et al., 2004)

If women did remain, they drew ‘attention’ when attempting exercises not generally used by women (Coen et al., 2018). Some women also experienced unwanted attention from men, describing it as an ‘occupational hazard’ and occasionally receiving ‘unsolicited advice’ which prevented them from persisting with ST out of fear of criticism from men (Coen et al., 2018).

‘Usually it’s guys who will come up and like say, “don’t do it that way”, … I think is the reason why women don’t like to go lifting because they’re scared that … guys are gonna be scrutinizing them and like criticizing their techniques and go and give unsolicited advice.’ (Coen et al., 2018)

Some ST women experienced verbal discouragement from other male gym users, particularly female bodybuilders experienced received comments about their physique being attained through the use of steroids.

‘As soon as you walk into a gym you are looked at and, as soon as you start picking up weights, the guys feel intimidated…They will come along and misload the bar, [or say] “That’s too big for you.” Watch out you might hurt yourself. The girls’ weights are over there. That kind of thing. It is just totally disparaging of your sexuality. They are men, so for them it’s for real, but because you’re a girl you are just playing and they never take you seriously. They will say “Oh she’s on steroids”.’ (Bopp et al., 2004)

Whilst ST, some women felt like they were a ‘nuisance’, felt obliged to compromise the pace of their workout in deference to others by staying ‘over out of their [men’s] way’, not be ‘holding up equipment’, and almost ‘apologizing’ for ST (Coen et al., 2018). This often resulted in women feeling like their workouts were ‘rushed’ (Coen et al., 2018). Despite equipment being used by others, women felt the ‘pressure to get off’ equipment by men (Coen et al., 2018). Women felt they lacked confidence compared to males in gyms and felt unable to ‘call out’ men being disorganised or disruptive whilst ST without receiving negative comments such as ‘bitch’ in return (Coen et al., 2018). If conflict occurred during their ST, some women avoided confrontation entirely.

‘[Male] moved my weights & workout bench to the side & arranged the area for himself. I approached & stated I was partway thru a circuit—his response was he was on a time limit & his workout was more important than mine could possibly be—to find another area of the gym to do my girl exercises in … Rather than have a huge scene—I moved to a different area.’ (Coen et al., 2018)

#### Lack of Support

Unsupportiveness, discouragement, and gender-based stigmas from family members, friends, and significant others were also commonly stated barriers for lack of participation in ST. Some family members questioned what women were ‘trying to prove’ by ST (Bopp et al., 2004).

‘“You need to lose your weight … not bulk up”.’ (O'Brien et al., 2008)

Some family members expressed very strong and offensive negative feelings of the appearance towards female bodybuilders.

‘Female bodybuilders look sick and repulsive. They are transsexuals. Why does anyone want to look like that? Who finds female bodybuilders attractive? Gay men? Lesbians? Who? (asked by a brother of a female bodybuilder)’ (Bopp et al., 2004)

Some women found integrating with social gatherings challenging where family members would be unsupportive of their lifestyle.

‘Family meals and get-togethers have become a nightmare. I avoid them whenever I can … They expect me to eat the fatty foods that they prepare and feel rejected if I bring my own and yet I never lecture them to eat more healthily because they are overweight …they won’t accept my lifestyle choice at all … they seem to think I’d be happier if I got married, settled down and had children.’ (Bopp et al., 2004)

### Psychological Barriers

#### Lack of Progression of Unexpected Results

Not seeing progress at the expected rate and unexpected results were a frequently stated reason for discontinuing ST. Inability to ‘see results straight away’ (Fleig et al., 2016) was also another barrier for ST.

‘I feel bulk[y] . . . I feel like I'm hard and fat, not lean and stronger.’ (O'Brien et al., 2008)

Women who were overweight or obese generally expected weight loss after ST and felt particularly demotivated to continue ST if their weight was increasing despite knowing this may be a result of increasing muscle mass.

‘You might actually gain it [weight] because you might be gaining muscle … you might give up after that ... I can’t do this, I can’t be bothered.’ (Guess, 2012)

#### Interest, Personality, and Preference

Lack of enjoyment or being ‘lazy’ were some other reasons stated for not undertaking ST (Doherty et al., 2018). Some women felt that ST was ‘boring’ and ‘repetitive’ (Doherty et al., 2018). Personal preference, choosing to not make ST ‘a priority’ despite the availability of time, was another barrier to ST (O'Brien et al., 2008). Women who were overweight or obese commonly stated not feeling ‘comfortable’ or feeling too ‘self-conscious’ about exercising in a gym as a reason for not ST (Guess, 2012).

‘I would rather work indoors, put some music on and work out myself. ‘Cos I am too self-conscious to go to the gym with all these skinny little women’. (Guess, 2012)

#### Knowledge

#### Inadequate Knowledge and Understanding

Not knowing ‘how to lift weights’ (Bopp et al., 2004), ‘what to do’ (Nazaruk et al., 2016), or ‘which ones to do’ (Doherty et al., 2018) were frequently noted ST barriers, particularly in older adults. Some women displayed a poor understanding of the purpose of various ST exercises.

‘Not 100% sure if I know exactly what is meant by muscle strengthening exercises’ (Doherty et al., 2018)

Other reasons for creasing ST were ‘remembering’ (Fleig et al., 2016) how to perform the exercises or ‘how to use them [equipment]’ (Guess, 2012). Lack of knowledge of ST meant women resorted to aerobic exercises because it was ‘easier’ (Guess, 2012).

#### Misconceptions and Gender-based Stigmas

Some comments were based on gender-based stigmas such as thinking women ‘don’t really need weights’ (Guess, 2012).

‘I do not think it [ST] plays as big a role in women’s health as it does in men’s …’ (Nazaruk et al., 2016)

Women also stated some misconceptions such as ST only improves the ‘look of your body’ (Nazaruk et al., 2016) and not health and that discontinuing ST can result in muscles turning into ‘flab and fat’ (Doherty et al., 2018). Women also commonly experienced misconceptions of what women’s bodies can look like after ST.

‘Nine times out of ten all the people I talk to and say ‘I’m a weightlifter’, they say ‘Oh, you don’t look like a weightlifter’. ‘What do you think a weightlifter looks like?’ [They say], ‘Someone that’s big and muscly and fat, more or less.’ I say, ‘That’s just the problem.’ We have girls that are World Champions that weigh 43 kg that lift enormous weights. They just have no idea because we have had people like Bev Francis and Gail Martin who have set the ball rolling with bigness associated with weightlifting.’ (Bopp et al., 2004)

### Physical Barriers

#### Injury, Pain, and Inadequate Fitness

Injury and perceived risk of injury due to incorrect technique were a prominent barrier against starting ST across multiple studies. Older adults were especially concerned about the ‘limitations of their body’ and questioned their ability to carry out strenuous activity (Dodd et al., 2006). Some exercises were perceived as ‘bad’ for certain body parts (Dodd et al., 2006), and some women were concerned about possible worsening of existing health conditions if they engaged in ST.

‘I would imagine I would get back problems … if I was doing anything very strenuous [ST].’ (Doherty et al., 2018)

Post-ST pain deterred many women were also deterred from ST across multiple studies. Women said that they would participate in ST if they were ‘fitter’ when ST would not ‘hurt as much’ (Doherty et al., 2018).

### Gym Infrastructure and Financial Barriers

#### Facilities and Accessibility

Some women emphasised the unavailability of ST equipment in their ‘community’ (Bopp et al., 2004), not having suitable equipment at home and ‘travelling to the likes of a gym’ (Doherty et al., 2018), as barriers to ST. Disabled women particularly highlighted the poor accessibility and inclusivity of many gyms.

‘[gym are] still not … accessible... access only relates to the frontage… How is that inclusive if you don’t provide a toilet for someone? … Your money isn't as valuable as the next persons.’ (Richardson et al., 2017)

#### Financial Barriers

Women also noted the ‘financial barriers’ (Dodd et al., 2006) of ST such as having to purchase equipment or a gym membership and would continue if they ‘could afford it’ (Dodd et al., 2006). Older adults also emphasised how expensive powerlifting programmes with supervised instruction were.

‘It was much more expensive than anything else I’d done before and that was one of my anxieties at the time.’ (Dodd et al., 2006)

#### Supervision and Instructors

A repeatedly stated barrier for ST was lack of instructors or supervision. In particular, a strict, ‘pitbull instructor’ who monitored gym attendance, and ‘guilt-tripped’ them into ST provided women with accountability and ‘inner motivation’ to engage in ST (O'Dougherty et al., 2008). The lack of a pre-determined schedule, excessive flexibility, and no expectation of attendance was, instead, a reason for discontinuing of ST.

‘If it’s set and you expect me to be there, then I will be there. But if you leave me to create my own timeline, what begins as Monday will ultimately end up Friday.’ (O'Brien et al., 2008)

Older people also stressed the importance of a good instructor in maintaining their adherence to ST, particularly due to a fear of injuries.

‘…I know it does happen, and people do injure themselves, so, for me, that’s really important to have someone supervise every movement...’ (O'Brien et al., 2008)

### Time-Effort Barriers

#### Availability of Time and Work-Life Balance

Lack of time was a reoccurring barrier to starting or maintaining ST even doing home workouts. Travelling for work and not being at home for extended periods was challenging to fit around a ST schedule and describing it as ‘too much’ (Tulloch et al., 2013).

‘Now my work schedule is, I travel to [locations], so I have a very heavy schedule, …didn’t expect all of this … It’s more demanding. I am gone one and a half weeks every month’ (Tulloch et al., 2013)

Working women highlighted the lack of ‘motivation’, being ‘exhausted’, ‘energyless’, and having to come home and ‘cook and do the dishes’ as reasons for disengagement in ST (O'Dougherty et al., 2008). Women mentioned that having a ‘regular schedule’ made ST easy to maintain; however, holidays or loss of routine were contributors to cessation of ST (O'Brien et al., 2008).

### Family Commitments and Other Obligations

‘Family constraints’ (Doherty et al., 2018) were also commonly stated as reasons for poor engagement in ST. Some who were caregivers or had other obligations even described managing their time and trying to incorporate everything as ‘overwhelming’ (O'Brien et al., 2008) and ‘unrealistic’ (Fleig et al., 2016) and often having to work around others’ schedules (O'Brien et al., 2008).

‘I enjoy coming, it's dealing with all the other people and things going on around you, it's like they tug on you and keep you from coming.’ (O'Brien et al., 2008)

Some women felt there was a lot going on in their lives which demotivated them from ST.

‘The biggest barrier has been the stressors in my life, but I usually go, so I haven’t let them be barriers, but certainly challenges. There are some days when your brain just doesn’t want to know about it. You have to work quite hard to be focused…if I’m not doing so well…and we’re in a stressful period, the hardest thing for me is to actually focus and switch off for an hour and not be distracted by those things’ (Dodd et al., 2006)

### Motivators

The papers contributing to each theme of motivators are shown in Table 4.

**Table 4:** How each study contributed to each theme—motivators

	**Social**	**Psychological**	**Gym infrastructure and financial motivators**	**Knowledge**	**Previous ST**
Bopp et al. (2004)		Yes		Yes	None
Brace-Govan (2004)	Yes	Yes	Yes	Yes	Elite athletes
Coen et al. (2018)		Yes		Yes	Regular ST
Dionigi (2007)	Yes	Yes		Yes	No ST (otherwise active)
Dodd et al. (2006)	Yes	Yes	Yes	Yes	Unknown
Doherty et al. (2018)	Yes	Yes		Yes	70% no ST 20% some ST 10% regular ST
Fleig et al. (2016)	Yes	Yes	Yes	Yes	Unknown
Foyster et al. (2020)	Yes	Yes	Yes	Yes	Regular ST
Gilson et al. (2008)	Yes	Yes			Structured ST up to 5 x per week
Gluchowski et al. (2018)	Yes	Yes		Yes	ST intervention
Guess (2012)	Yes	Yes	Yes		Unknown
McGlashan et al. (2020)	Yes		Yes	Yes	Regular ST
Nazaruk et al. (2016)		Yes		Yes	Unknown
O'Brien et al. (2008)	Yes				Unknown
O'Dougherty et al. (2008)	Yes	Yes	Yes		Sedentary–modestly active
Richardson et al. (2017)	Yes	Yes			Unknown
Rosenthal et al. (2021)	Yes	Yes		Yes	Regular ST
Shilling and Bunsell (2009)		Yes			Regular ST
Tulloch et al. (2013)	Yes	Yes	Yes		No ST (sedentary)
Worthen and Baker (2016)	Yes	Yes		Yes	Regular ST

### Social Motivators

#### Camaraderie in Training

Women emphasised the social aspect of ST across multiple studies. Women mentioned that the group they were with during the period of the study provided them with ‘encouragement’ and ‘support’ to push themselves and continue (Dodd et al., 2006). This sub-theme was particularly prevalent amongst women with health conditions and older women, where being surrounded by other women of a similar background to them created a supportive and ‘comfortable’, ST environment (Dodd et al., 2006).

‘If you’ve got MS you … underestimate your abilities … you need a fair amount of encouragement . . . it was nice to have people who didn’t seem to focus on the fact we … have an illness.’ (Dodd et al., 2006)

‘I really do enjoy seeing those who are (competitors), and who are enjoying that-that aspect of it. It’s interesting to see, and . . . but by the same token, that’s . . . it’s also more comfortable and reassuring to be in a group of women . . . older women, and a few of the men as well, um, who are also dealing with an aging process. Um, that feels more comfortable.’ (Dodd et al., 2006)

Older women, women with disabilities, MS, and Parkinson’s disease particularly highlighted the benefit of friendship, and prior arrangement of ST encouraged them to attend.

‘We’ll [friends made in the gym] meet up and somebody will say “I’ll see you next week then?” … “I don’t want to let them down so I’ll go.” … It builds up this peer support…’ (Richardson et al., 2017)

In contrast to barriers, some women who were overweight or obese noted that they ‘feel better’ ‘meeting people and the instructors’ (Guess, 2012) rather than ST alone. Women mentioned that it ‘gets [them] out’ (Guess, 2012), encourages them to push themselves harder and benefit from group discussions and questions asked by others.

‘It was a little bit competitive … you were still sort of watching other people, saying well they’re getting along further than I am. I might need to try a little bit harder’ (Guess, 2012)

Older women emphasised interactions with younger people and how it motivated them to engage in ST.

‘I enjoyed … the contact with the young people … intergenerational meeting … was one of the plusses.’ (Dionigi, 2007)

Women currently meeting ST recommendations mentioned that their main motivator to participate in ST was their team.

‘[The team] that you’re doing it for …. it [ST] makes my team better.’ (Gilson et al., 2008)

#### Support From Friends, Family, and Significant Others

Women also mentioned that individuals such as family, friends, and healthcare professionals motivated them to participate in ST. Uplifting comments from those around them were also strong motivators.

‘Oh, you look good, like you're getting into shape.’ (O'Dougherty et al., 2008)

Family and friends reminding women to train or ‘doing it [ST] together’ (Tulloch et al., 2013) was another motivator that was frequently mentioned across studies.

‘I get my husband to drag me out of bed in the morning. It’s helped that he’s going [to the gym] with me, so it’s like a combined effort.’ (Tulloch et al., 2013)

Some women mentioned that their motivator for undertaking ST was because they witness people around them engaging in ST and decided to ‘follow the trend’ (Doherty et al., 2018).

‘Oh, I bumped into a- a friend, who was, um, doing powerlifting. And she’d just come from the gym-… and she was raving about it. So, I thought, oh wow. And I got my husband, and I said, ‘Let’s go and have a try out day.’’ (Dodd et al., 2006)

Female athletes were motivated to participate in ST by their competitors who are ‘a lot bigger [and] stronger’ or coaches training them (Gilson et al., 2008).

Women were also motivated to participate in ST by hearing other women’s success stories or ‘incorporating their ideas’ into their own training (Fleig et al., 2016).

### Psychological Motivators

#### Body Image and Progression

Body image and improving physique were also a frequently noted motivator for starting strength training. Women stated that strength training made their physique ‘more attractive’, ‘more feminine’, and ‘tone[d]’ (Nazaruk et al., 2016).

Some women undertaking regular ST used the gender-stigma in a positive light.

‘Being strong was a part of it [her goal] but I still like the concept of having a big, strong body. I was proud of it. I worked hard for it. If somebody mistook me for a man, which often happened, I felt good about it because they were acknowledging that I had a big strong body. Others felt offended. Why be offended? You have just broken a stereotype and you have got to expect people to react in that way.’ (Bopp et al., 2004)

Other women mentioned ST also gave them ‘confidence’ and they felt ‘empowered’ (Worthen & Baker, 2016). Older women and women with MS particularly felt more ‘normal’ (Dodd et al., 2006). ST also instilled confidence in some women to push themselves further.

‘They [women] feel like they can do more … because they lifted some weights … they just feel that confidence…’ (Nazaruk et al., 2016)

Women also conveyed across multiple studies that visible progress was also a motivator to continue ST.

‘Seeing the difference between how I am now and how I was at the beginning.’ (Dodd et al., 2006)

Across multiple studies, improvements in strength and ‘feeling stronger’ were identified as reasons for ongoing ST (O'Dougherty et al., 2008). Women who were overweight or obese were particularly motivated by reducing numbers on the scales, tracking their progress and seeing ‘the quickest benefits’ such as improved muscular tone (Guess, 2012).

‘Looking at the scale … I am losing … I need to keep going, it’s motivating to me.’ (Guess, 2012)

Older people often stated that measurements of their progress motivated them; some forms of progression included bone density scans.

‘I’d like to be measured again at the end of the next eight weeks, the measurement part is a huge motivator.’ (O'Dougherty et al., 2008)

‘I’ve had bone density scans because I had the condition for a long time and I’m thrilled to bits, first time ever, it increased, not a lot but it did increase. That’s been the biggest thing for me, really exciting.’ (O'Dougherty et al., 2008)

Older people frequently highlighted the changes in their activities of daily living after engaging in ST.

‘I can squat down now, and I can just get up again, which is fabulous. I can go up an incline…I can go up and down steps now and I don’t need to hang onto anything’. (O'Dougherty et al., 2008)

#### Sense of Obligation

Some women were motivated because they felt obliged to complete the ST intervention part of the study. Others noted reasons such as being ‘dedicated’, not ‘cheating’ (O'Dougherty et al., 2008) themselves or the study and being ‘self-motivated’ (Dodd et al., 2006) to continue ST.

‘I made a commitment to do it, signed an obligation, didn’t occur to me not to come; I signed up for the program, like I paid for a class at a fitness club.’ (O'Dougherty et al., 2008)

#### Mental-Health Benefits

Many women across multiple studies quoted they were ‘feeling better’ (O'Dougherty et al., 2008), ‘happier’ (Dodd et al., 2006), and ‘invigorated’ (Dionigi, 2007) had feelings of ‘accomplishment’, ‘higher energy levels’ (Dodd et al., 2006) and were ‘more aware and alert’ (Doherty et al., 2018) as motivators for ST, particularly older women and those with other health conditions.

Women who were athletes or bodybuilders stated reasons such as being ‘competitive’ (Worthen & Baker 2016) or an ‘overachiever’ (Gilson et al., 2008), wanting ‘to win’ (Gilson et al., 2008), and having a desire to challenge themselves ‘physically’ (Worthen & Baker, 2016) which motivated them to continue ST.

### Knowledge

#### Improvement of General and Musculoskeletal Health

Many women who had knowledge of the physical (especially musculoskeletal) and mental-health benefits of ST, indicated this knowledge as their motivating factor for taking part in it. Older women or those with health conditions felt that ST improved their symptoms, daily living, and was ‘delaying or prolonging’ (Dodd et al., 2006) disease progression which motivated them to continue ST.

#### Increased Knowledge and Understanding of ST

Increased knowledge of ST, its benefits, and the correct techniques encouraged some women to begin ST.

‘I have a more open mind now. To the weights. Given the benefits involved. I never knew that… before I was drummed into the aerobics…’ (Guess, 2012)

For younger participants, it was important to change parental misconceptions to enable access to weight training.

‘I asked them [the girls] to bring their parents in so that I could have a chat to them about women weightlifting and I am sure a lot of the Mothers coming in thought they [the girls] would get big and have lots of muscles and that it was going to injure them in some way – sure 90% came in thinking that way and changed their mind when they left . . . I certainly changed a few Mothers’ ideas.’ (Dodd et al., 2006)

### Gym Infrastructure and Financial Motivators

#### Financial Incentive

Ease of access to the gym, ‘the facility’ (Dionigi, 2007), and financial incentives (O'Dougherty et al., 2008) were commonly stated reasons for motivation for continuing in ST.

#### Supervision and Instruction

Women also highlighted the benefit of having an instructor providing them with guidance, ‘accountability’, and progress tracking prevented women from giving ‘excuses to skip and change’ (Tulloch et al., 2013).

Older adults especially stressed the importance of their instructors in motivating them to engage in ST.

‘I knew [the coach] thought I could do it and I wanted to do it, so no I wasn’t scared or nervous because he talks you into it and I thought, “Now this is going to be hard,” and it was. And I was able to do it’ (Dodd et al., 2006)

## Discussion

In this exploration of women’s initiation and maintenance of strength training, the main barriers and facilitators were social factors and the most commonly explored factors were perceptions of gender-based stigma. When friends and family supported women by praising them and accompanying them to the gym, this motivated women to continue ST; however, when friends and family discouraged them, they found it challenging to continue ST. The results of the training also influenced maintenance of ST. Where women saw progress, this was motivating, but when progression was slow or unexpected, this was a barrier. Other factors associated with poor adherence included boredom and repetition of ST exercises, poor knowledge of ST, poor accessibility in gyms, lack of supervision or routine, and difficulty in balancing work, time, family life, and other commitments with ST.

### Fit with Previous Research

Previous research, particularly older studies such as Harne and Bixby (2005) found time-effort barriers were most prevalent; however, we identified social barriers such as gender-based or social stigmas and lack of social support to be most prominent which is more consistent with newer research. (Peters et al., 2019; Burton et al., 2017; Cavill & Foster, 2018) Although these studies identified similar social barriers, this review particularly provided greater detail and understanding of why women felt uncomfortable in ST environments. In particular, being perceived as ‘princesses’, receiving negative comments, drawing attention, and feeling self-conscious exercising with others provided greater insight into why evaluation concerns (an individual’s interest in others’ opinion of them) are more common in women as shown by Salvatore and Marecek (2010).

In ST, competency and skill are evident to bystanders and are assessed by observing technique and physique. This is particularly challenging for individuals who are overweight or obese and can lead to feelings of self-consciousness. Unwanted comments, perceptions, and gender-stereotyping may therefore spark evaluation concerns about incompetency and initiate a self-perpetuating cycle where evaluation concerns prevent women from using ST equipment and improving proficiency. This leads to a lack of development in skill and strength which results in additional evaluation concerns, further decreasing ST participation and possibly avoiding exercise environments altogether. This makes it even more challenging for previously sedentary women to engage in ST as shown by Salvatore and Marecek (2010)

The segregation of cardiovascular and ST equipment has not been mentioned in other studies as a barrier to women’s participation in ST. The cardio area was perceived by some women as ‘their place’ at the gym and the lack of ST equipment was sometimes seen as women not requiring the weights. Johansson (1996) also highlighted the issue of ‘gendered spaces’ in relation to the ST regions of the gym. Although these areas are not exclusively male territory, entering this region can make women often feel alien and crowded out, particularly disproportionate use of space and equipment by men both physically and sonically through noise such as ‘grunting and groaning’. Women experienced in ST also raised the issue of an unsaid ‘pecking order’ where stronger people appear to have ‘more right’ over the equipment and are able to assert themselves over equipment ((Bopp et al., 2004)). This is generally the case for both men and women; however, this tends to especially intimidate women at the start of their training. Such ST environments in combination with unsupportiveness, discouragement, and gender-based stigmas from friends and family strengthen the gender-typing of ST and further decrease uptake.

Many of the psychological, knowledge, physical, gym infrastructure, and time-effort barriers and motivators identified in this review have also been reported in other studies (Peters et al., 2019; Hurley et al., 2018; Harne & Bixby, 2005; Burton et al., 2017; Cavill & Foster, 2018; Rhodes et al., 2017); however, this review identified some specific factors in detail that were psychological demotivators for ST and particular patterns that arose in women who engaged in ST regularly compared to women who did not engage in ST regularly. For example, desire to see progress quickly or immediately and unexpected results such as weight gain particularly in women who were overweight or obese as shown by Guess (2012) were specific to ST. Improvements in bone density and activities of daily living were strong motivators for older women. Some women also mentioned that they were motivated to train, because they saw others do the same, and others identified the need to have a strict instructor that provided accountability and guilt to motivate them to undertake ST. Women who strength trained regularly noted social and psychological barriers more often, particularly the social barriers. Those who did not engage in ST highlighted the time-effort and physical barriers as reasons for not undertaking ST. An additional overarching issue for these women could be seen as ‘misinformation,’ such as not being aware of the potential benefits for women, and not knowing how or where to start with training. Older women generally highlighted physical and financial barriers. Social and psychological motivators were present across all women regardless of previous ST experience.

Understanding how these barriers and motivators fit together may be facilitated by the use of a theory of behaviour change such as the self-determination theory (SDT) or the social cognitive theory (SCT). For example, SDT can be applied to some of the respondents in this review, where motivation to achieve the same behavioural goal (e.g. weight loss, diet, and exercise) can be externally driven (e.g. to avoid criticism from healthcare or fitness professionals), partly self-regulated (e.g. following the trend), all the way to autonomous self-regulation (e.g. keep charts, logging progress) (Teixeira et al., 2012; Baumeister et al., 2006). The SCT, on the other hand, offers a theoretical approach to ST maintenance whilst taking into account the dynamic nature of ST, self-regulation strategies, change in mood after ST, and environmental motivators and barriers to ST (Winett et al., 2009). Future research should examine these themes through the lens of a behaviour change theory, and these theories should then be used to inform which interventions are developed and implemented.

This review also identified a poor understanding of the purpose or meaning of ST and some misconceptions amongst women which had not been reported in other studies or reviews. Abula et al. (2018) and Prochaska and DiClemente (1983) both showed that simply providing knowledge of guidelines is insufficient to induce a more active lifestyle and that further education of health benefits is necessary to increase uptake. In this review, when some women with poor prior knowledge of ST were provided information about its benefits, this encouraged them to try ST. A plausible explanation for this may be that, although guidelines are available online, this may be inaccessible to some individuals and may mean that addressing these issues in clinical or exercise settings is key to improving uptake of ST.

We also particularly found that group ST programmes encouraged women to push themselves, form new friendships or peer support, and made women feel encouraged to attend sessions they had signed up to or paid for. Furthermore, supportive family and friends who reminded women to train, trained with them, and offered words of affirmation were social motivators. Box et al. (2019) also showed that affiliation and competition with other exercise attendees increased as the length of time of training increased, possibly due to increased ability, self-efficacy, and comfort within the exercise environment to perform at a competitive level (Ryan Shuda & Feito, 2017). In this review, further increase in comfort from having family and friends around during exercise could also be an explanation for this being a strong motivator to maintaining ST. This was also consistent with Ryan, Shuda and Feito (2017) and Heinrich et al. (2017) who demonstrated the importance of social and environmental factors for individuals engaging in high-intensity functional training (HIFT) on adherence. HIFT can be compared to ST since it uses bodyweight exercises and resistance to improve muscle mass and strength. The greater length of participation in HIFT meant that increased comradery and self-efficacy, as well as the competitive nature of HIFT, motivated individuals to continue. Thus, social support, comradery, self-efficacy, and friendly competition may be especially important in ST given its high-intensity nature, to encourage women to push themselves and build up the support network to continue training in the future.

### Strengths and Limitations

Some limitations of the search and selection process include that only three databases were searched, although forwards and backwards searching picked up a number of other papers. Our main limitation was that, due to resource issues, only one author screened all titles and abstracts, assessed study quality, and coded data from papers; however, this was done with close supervision and regular checks with the second author. The Joanna Briggs Institute Checklist for Qualitative Research was also used to appraise both qualitative and mixed-methods studies which may have not provided the most robust quality appraisal for mixed-method papers.

The main strengths of this review were that women were sampled from a range of different backgrounds, health conditions, and ages, reducing selection bias. Although women from these backgrounds have different motivators and barriers to those without named conditions, this approach provided a fuller overall picture of the motivators and barriers to ST in women. Furthermore, the meta-synthesis provided a comprehensive and detailed understanding of the barriers and motivators of strength training through direct quotes about women’s experiences. Many of these factors mentioned would have been difficult to assess using purely quantitative methods.

Limitations of literature identified for the review included that the sample of women obtained were all from developed western countries with a predominantly Caucasian background despite some studies including samples of women from different ethnic backgrounds. Exercise rates in general are lower amongst those from ethnic minority groups, particularly Asians (Saffer et al., 2013), and qualitative research identifying motivators and barriers for strength training in these groups needs to be addressed in future research, possibly due to language barriers as identified in previous studies. Further research could also focus on asking women where they get their main exercise information from, for starting an exercise programme, which would enable interventions directed to aspects of knowledge gathering that need addressing. A sensitivity analysis of difference in motivators and barriers for healthy women and those with pre-existing health conditions would also help direct the right interventions towards the right target group. It may also be helpful to investigate further the benefits of exercise that are not visible or able to be tracked with the eye, such as HbA1c, cholesterol, muscle mass, as it was seen in one study that increasing bone density was a great motivator (Gluchowski et al., 2018). Tracking these markers in addition to weight, perceived body fat, and measurements could be a beneficial addition to future programmes.

### Impact of Review on Prevention Strategies

Engaging more women in ST may help prevent a range of conditions. Physical inactivity is a significant predictor of cardiovascular disease, type 2 diabetes mellitus, obesity, cancer, musculoskeletal conditions, depression, and overall mortality (Hallal et al., 2012), suggesting that promoting physical activity, particularly ST, will reduce women’s risk factors for these conditions. However, ST remains a minority activity for women, and this review offers an insight into potentially effective strategies to increase uptake.

Training healthcare professionals (HCPs) and fitness professionals to deliver ST advice and education to men, women, and families in clinical, community, or exercise settings may help reduce lack of support, gender-based stigmas, and misconceptions and increase knowledge of meaning, purpose, and benefits of ST, thus enabling ST to be used as prevention strategy for musculoskeletal and metabolic conditions, which are becoming more prevalent. Information and advice could also be provided through trusted websites (NHS UK or public health websites) or leaflets that HCPs can offer patients who seek more information. An exercise prescription or referral service for personal training may also ease transition from a sedentary to a healthier lifestyle and has been trialled by some UK public health teams. Future engagement programmes need to acknowledge that there is a lot of misunderstanding or false information about ST, and programmes may need to start at this position to address these false beliefs. Engaging with potential end-users to co-design the program being offered may help overcome this barrier.

Offering women-only ST programmes or ST areas or times, mobile phone applications to indicate numbers of peoples currently at the gym and group personal training sessions with female trainers may help reduce evaluation concerns, help women become more accustomed to the ST environment, see female trainers as role models (Lockwood, 2006), and help promote a more gender-neutral and approachable gym environment. It will also help counteract knowledge barriers such as poor technique and difficulty remembering exercises, as well as offering initial accountability, routine, confidence, and encouragement to those who need it.

Furthermore, trainers should inform women on realistic rates of progression, appropriate measurement of progress, possible physical consequences such as pain or injury, and advice on management or seeking professional help. Offering alternative methods of assessing progression such as measuring waist circumference or body fat percentage may be a useful tool in counteracting psychological barriers and promoting motivators particularly in women who are overweight or obese who are especially demotivated by weight gain.

Making ST programmes less repetitive (i.e. incorporating circuit training), more engaging, more family-friendly, and incorporating group training may help counter psychological barriers such as boredom and boost the social motivators. Easy access to reasonably priced crèche services may also help overcome the barrier of family obligations and having to work around others’ schedules. Working women who travel may especially benefit from gym memberships offering access to multiple gyms nationally. Provision of online or verbal advice on meal preparation and planning via health promotion websites, online videos, tutorials, or classes may help free up time and energy for ST during the week.

An initiative to provide free or subsidised gym memberships, initial personal/group training sessions, or home bodyweight ST workouts using minimal/accessible equipment may help alleviate the accessibility and financial barriers of ST for those from low socio-economic groups. Inadequate disabled access was also a significant barrier highlighted by disabled women which is a factor that needs to be addressed by fitness centres and gyms.

The factors identified in this review may help researchers, policymakers, public health and health promotion strategists, recreation programmers, and healthcare and fitness professionals to develop their understanding of how to help women engage in ST and reduce their risk of a range of conditions. Using theory-based interventions/approaches to address barriers to ST may enable delivery of more effective advice and encouragement in healthcare or exercise settings and help improve existing or future ST programmes and gym or fitness centre environments in a way that encourages more women to participate in ST. It may also be beneficial in integrating ST programmes into NHS and other healthcare services.

Increasing engagement with ST could particularly help prevent osteoporosis and related falls, providing healthcare systems with a financial incentive to implement such interventions to help reduce the increasing burden of osteoporosis-related admissions. Osteoporosis is much more challenging to tackle after it has already developed, and thus, applying strategies to prevent it from occurring in the first place is necessary. Since consistency has been shown to be key in developing lean muscle mass and improving strength (Coffey et al., 2006), promoting the motivators and placing suitable strategies to address the barriers to women’s participation in ST is crucial to improving adherence and thus, female musculoskeletal health.

## Conclusions

This study identified key barriers and motivators for women to taking up and maintaining strength training, including social, knowledge, and time-effort barriers. We propose a number of ways HCPs, communities, gyms, and fitness professionals could address the barriers and improve women’s uptake of ST. Incorporating efforts to reduce prevalent gender-based stigmas around ST into gym design, as well as class and fitness programme structures would help to make women feel more welcome in gym environments. Making ST programmes group-based, less repetitive, family-friendly, offering choice of home workouts, subsidising gym memberships, and improving disabled access will help reduce the psychological, time-effort, and extrinsic barriers.

Increasing women’s access to ST will enable substantial improvements to their musculoskeletal and metabolic and mental health, especially as they age. Training habit, confidence, and knowledge are built over time and addressing the issues for younger as well as older women will enable more women to take up ST earlier in life and continue ST later in life, helping to increase women’s quality of life and to reduce the burden of musculoskeletal disease on health systems.
